# Lipid rafts enriched in monosialylGb5Cer carrying the stage-specific embryonic antigen-4 epitope are involved in development of mouse preimplantation embryos at cleavage stage

**DOI:** 10.1186/1471-213X-11-22

**Published:** 2011-04-14

**Authors:** Ban Sato, Yohko U Katagiri, Kenji Miyado, Nozomu Okino, Makoto Ito, Hidenori Akutsu, Hajime Okita, Akihiro Umezawa, Junichiro Fujimoto, Kiyotaka Toshimori, Nobutaka Kiyokawa

**Affiliations:** 1 Department of Pediatric Hematology and Oncology Research, National Research Institute for Child Health and Development, 2-10-1 Okura, Setagaya-ku, Tokyo 157-8535, Japan; 2 Department of Reproductive Biology, National Research Institute for Child Health and Development, 2-10-1 Okura, Setagaya-ku, Tokyo 157-8535, Japan; 3 Department of Bioscience and Biotechnology, Graduate School of Bioresource and Bioenvironmental Sciences, Kyushu University, Hakozaki 6-10-1, Higashi-ku, Fukuoka 812-8581, Japan; 4 Department of Anatomy and Developmental Biology, Inohana 1-8-1, Chuo-ku, Graduate School of Medicine, Chiba University, Chiba 260-8670, Japan

## Abstract

**Background:**

Lipid rafts enriched in glycosphingolipids (GSLs), cholesterol and signaling molecules play an essential role not only for signal transduction started by ligand binding, but for intracellular events such as organization of actin, intracellular traffic and cell polarity, but their functions in cleavage division of preimplantation embryos are not well known.

**Results:**

Here we show that monosialylGb5Cer (MSGb5Cer)-enriched raft domains are involved in development during the cleavage stage of mouse preimplantation embryos. MSGb5Cer preferentially localizes at the interfaces between blastomeres in mouse preimplantation embryos. Live-imaging analysis revealed that MSGb5Cer localizes in cleavage furrows during cytokinesis, and that by accumulating at the interfaces, it thickens them. Depletion of cholesterol from the cell membrane with methyl-beta-cyclodextrin (MbCD) reduced the expression of MSGb5Cer and stopped cleavage. Extensive accumulation of MSGb5Cer at the interfaces by cross-linking with anti-MSGb5Cer Mab (6E2) caused F-actin to aggregate at the interfaces and suppressed the localization of E-cadherin at the interfaces, which resulted in the cessation of cleavage. In addition, suppression of actin polymerization with cytochalasin D (CCD) decreased the accumulation of MSGb5Cer at the interfaces. In E-cadherin-targeted embryos, the MSGb5Cer-enriched raft membrane domains accumulated heterotopically.

**Conclusions:**

These results indicate that MSGb5Cer-enriched raft membrane domains participate in cytokinesis in a close cooperation with the cortical actin network and the distribution of E-cadherin.

## Background

The molecular dynamics involved in embryogenesis is now being elucidated. In the early cleavage stage of embryogenesis, the localization of cell surface molecules periodically changes and is spatio-temporally controlled. Cytokinesis is a fundamental process of cell cleavage in which the daughter cells split after nuclear division, and it is driven by actin-dependent narrowing of a contractile ring as well as furrow-specific addition of membrane [1,2]. The latter contributes to dynamic rearrangement of cell surface proteins and provides molecules required to construct the complex machinery of cytokinesis. For example, the cell surface adhesion molecule E-cadherin is drastically rearranged in a close correlation with the dynamics of cortical actin [3]. It is well documented that E-cadherin is located predominantly in membrane domains involved in cell-cell contacts of adjacent blastomeres and mediates adhesion between blastomeres of preimplantation mouse embryos from 8-cell stage onwards [4-6].

A new aspect of the cell membrane structures called "lipid rafts" has been postulated [7]. Lipid rafts have been described by Lingwood and Simons as "fluctuating nanoscale assemblies of sphingolipid, cholesterol, and proteins that can be stabilized to coalesce into platforms that function in membrane signaling and trafficking" [8]. During cell cleavage in embryogenesis, lipid rafts were shown to play an essential role in central spindle assembly and cleavage furrow ingression [1]. For example, GM1 is a monosialylated ganglio-series glycosphingolipid (GSL), which is most commonly used as a raft marker. The lipid rafts enriched in GM1 at the cleavage furrow were shown to possess signaling machinery that contributes to cytokinesis during the cleavage of sea urchin eggs and mouse preimplantation embryos [9,10].

We recently reported finding that monoclonal antibody (Mab) 6E2 raised against human embryonal carcinoma cell line recognizes the globo-series GSL MSGb5Cer is present at the interfaces between blastomeres of living mouse embryos [11]. MSGb5Cer carries an epitope of stage-specific embryonic antigen-4 (SSEA-4), which expressed only in the early cleavage stage of mouse embryos. Lipid rafts enriched with MSGb5Cer was also reported to interact with E-cadherin in breast cancer cell lines [12]. Therefore, MSGb5Cer is expected to form lipid rafts on mouse preimplantation embryos and play an important role in the process of cleavage division. To elucidate the functional role of MSGb5Cer enriched-lipid rafts in embryogenesis, in this study we investigated the dynamics of MSGb5Cer in preimplantation embryos during the course of early embryonic cleavage.

## Results

### MSGb5Cer and E-cadherin localize at the interfaces between the blastomeres in mouse preimplantation embryos

**Figure 1:**
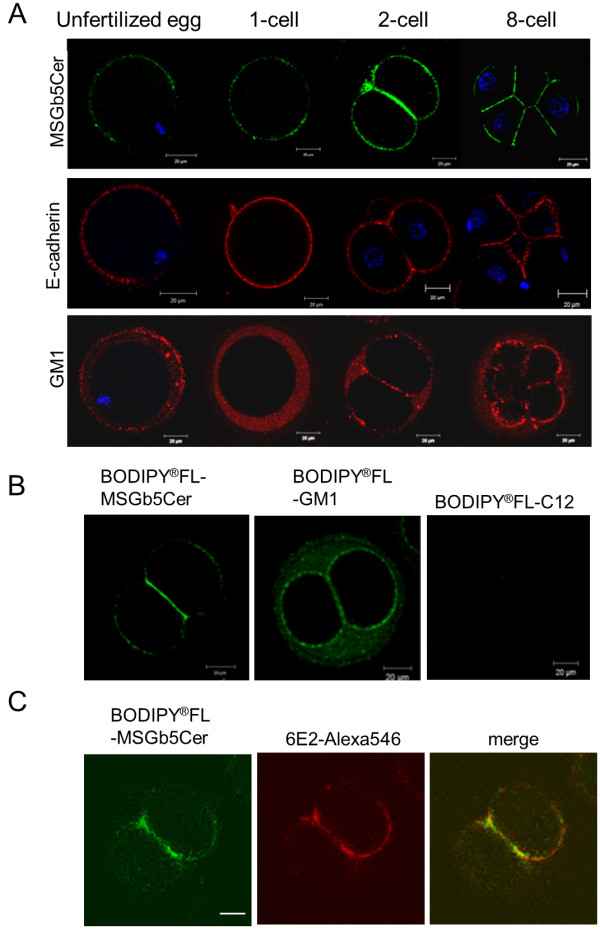
**MSGb5Cer localize at the interfaces between blastomeres in mouse preimplantation embryos**. (A) Localization of MSGb5Cer, E-cadherin and GM1 in an unfertilized egg and an embryo at the zygote, 2-cell stage and 8-cell stage. MSGb5Cer (top row), E-cadherin (middle row), and GM1 (bottom row) in a living mouse egg or embryos were stained with Alexa Fluor^® ^488-conjugated 6E2, ECCD-2/Alexa Fluor^® ^546 Rabbit Anti-rat IgG, and biotinylated-CTX B/Alexa Fluor^® ^546 streptavidin, respectively, and examined. Thirty sample unfertilized egg and 45 sample embryos of other stages were used for this experiment represented in image. (B) Distribution of BODIPY^®^FL-MSGb5Cer and BODIPY^®^FL-GM1 in a 2-cell stage embryo. The distribution of MSGb5Cer and GM1 is shown in the form of optical slice images. BODIPY^®^FL-C12 fatty acid was used as a negative control. All scale bars represent 20 μm. Ten sample 2-cell stage embryos were used for this experiment represented in each image. (C) Distribution of BODIPY^®^FL-MSGb5Cer in a 2-cell stage embryo cultured in the presence of 6E2. The BODIPY^®^FL-MSGb5Cer introduced 2-cell embryo was cultured for 1 hour in M16 medium containing 15 μg/ml of 6E2, and then stained with Alexa Fluor^® ^546-conjugated 6E2. Scale bar represent 20 μm. Ten sample 2-cell stage embryos were used for this experiment represented in image.

MSGb5Cer, E-cadherin, and GM1 were visualized in living mouse preimplantation embryos, and their localizations are shown in the optical slice images in Figure 1A. MSGb5Cer and E-cadherin were stained with Mabs 6E2 and ECCD-2, respectively, and they were localized on the cell surface in dotted form in unfertilized eggs but excluded from the area over the meiotic spindle. GM1, on the other hand, was stained with the cholera toxin B subunit (CTX-B) and had accumulated in a perivitelline space in unfertilized eggs, and its accumulation accelerated after fertilization. Preferential localization of MSGb5Cer and E-cadherin at interfaces between blastomeres was also observed in compacted 8-cell stage embryos, whereas GM1 mainly localized at the outer surface area and only a small amount of GM1 was detected at the interfaces. A part of MSGb5Cer was localized similar to GM1 at the outer surface area of blastomeres. These results indicate that MSGb5Cer and E-cadherin, but not GM1, are similarly distributed in preimplantation embryos.

Next, we enzymatically prepared fluorescence-labeled MSGb5Cer or GM1 and introduced into embryos instead of staining with GSL-specific Mab 6E2 or ligand CTX-B to visualize GSLs on embryos. As shown in the optical slice images in Figure 1B, BODIPY^®^FL-MSGb5Cer was distributed mainly at the interfaces between blastomeres, whereas BODIPY^®^FL-GM1 was evenly distributed over the surface and in the perivitelline space. In contrast, BODIPY^®^FL-C12 fatty acids, as a negative control, were not detected at all. The results coincided with that shown by immunostaining. The prolonged culture of BODIPY^®^FL-MSGb5Cer-introduced embryo in the presence of 6E2 at the same concentration used for immunostaining in Figure 1A did not affect preferential localization of MSGb5Cer, or did not induce the aggregation of MSGb5Cer (Figure 1C). Therefore, it is indicated that the preferential localization of MSGb5Cer at interfaces is due to spontaneous accumulation of MSGb5Cer at interfaces and is not due to the artifactual aggregation of MSGb5Cer induced by cross-linking with antibody.

### MSGb5Cer accumulates in the cleavage furrow of preimplantation embryos during cytokinesis, and then at the interfaces after cytokinesis

**Figure 2:**
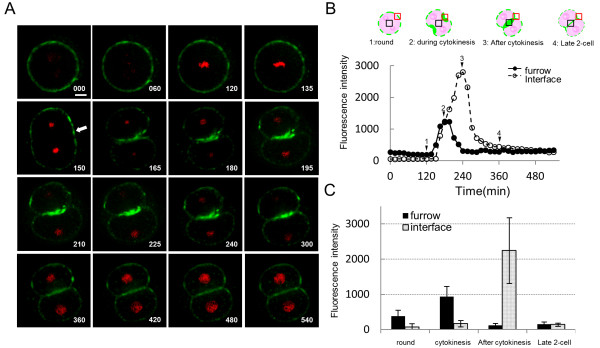
**MSGb5Cer accumulates at the interface during cytokinesis in preimplantation embryos**. (A) Frames from a series of confocal time-lapse movies of live cytokinesis-stage cells stained with 6E2 for MSGb5cer (green) and with DAPI for nuclei (red). Frames are shown at 60-minute intervals from 0 to 120 minutes, 15-minute intervals from 120 minutes to 240 minutes, and 60-minute intervals from 240 minutes to 540 minutes. Time is expressed in minutes after the start of culture. The arrow at 150 minutes points to the site of a future furrow in the spherical zygote. Scale bar represents 20 μm. The images shown are representative of nine sample zygotes. (B) Temporal changes in fluorescence intensity at the furrow and the interface region during cytokinesis. The fluorescence intensity in pixel values was measured in boxes of equal size located at the furrow (red square) and the interface (black square) of the frames taken every 15 minutes from a confocal time-lapse movie of the zygote of Figure 2A. The fluorescence intensity values at the furrows (closed circles) and interface (open circles) were plotted against time in minutes. Schematic embryos at the round stage (1), during cytokinesis (2), after cytokinesis (3), and the late 2-cell stage (4) were cultured for 105 minutes, 165 minutes, 240 minutes, and 405 minutes, respectively. This data shown is representative of nine sample zygotes. (C) The fluorescence intensity of MSGb5Cer at the furrow (closed column) and interface (dotted column) in round, cytokinesis, post-cytokinesis and late 2-cell stage embryos. Mean values and s.d. were calculated from the results obtained from the time-lapse movies of 6 embryos shown in Figure 2B.

Time-lapse images of MSGb5Cer visualized with MSGb5Cer-specific Mab (6E2) are shown in Figure 2A. MSGb5Cer began to accumulate at the site of the future furrow (indicated by the arrow) of the spherical zygote at 150 minutes, and accumulation later accelerated and peaked at 180 minutes. Accumulation at the interface also accelerated, and after peaking at 240 minutes, gradually decreased to the initial level at 360 minutes (Figure 2A, B; see the Movie in additional file 1). The means ± s. d. of the fluorescence intensities in the furrow and at the interface of 6 embryos in the round stage, during cytokinesis, after cytokinesis, and in the late 2-cell stage are shown in Figure 2C. MSGb5Cer accumulated in the furrow at the start of cytokinesis and moved to the interface as cytokinesis proceeded. After cytokinesis, the accumulation was gradually broken, and MSGb5Cer eventually became evenly distributed over the entire cell surface, including the interface.

### Overlapping distribution of MSGb5Cer with cholesterol

**Figure 3:**
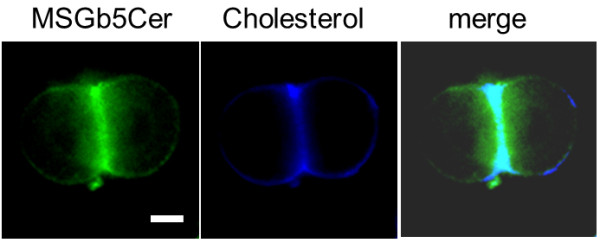
**MSGb5Cer is overlapped by cholesterol at the interface in a 2-cell embryo**. A two-cell stage embryo was stained with 6E2 for MSGb5cer (green) and then filipin III for cholesterol (blue) as described in Materials and Methods. Scale bar: 20 μm. Ten sample 2-cell stage embryos were used for this experiment represented in images.

Cholesterol is known to be an important constituent of lipid rafts. Since MSGb5Cer is thought to be also involved in the formation of raft membrane domains in preimplantation embryos, we examined the distribution of cholesterol with Filipin III in the 6E2-stained and fixed embryos. The merged images of MSGb5Cer and cholesterol showed overlapping localization at the interfaces between blastomeres (Figure 3).

### MbCD causes de-compaction and subsequent suppression of cell division

**Figure 4:**
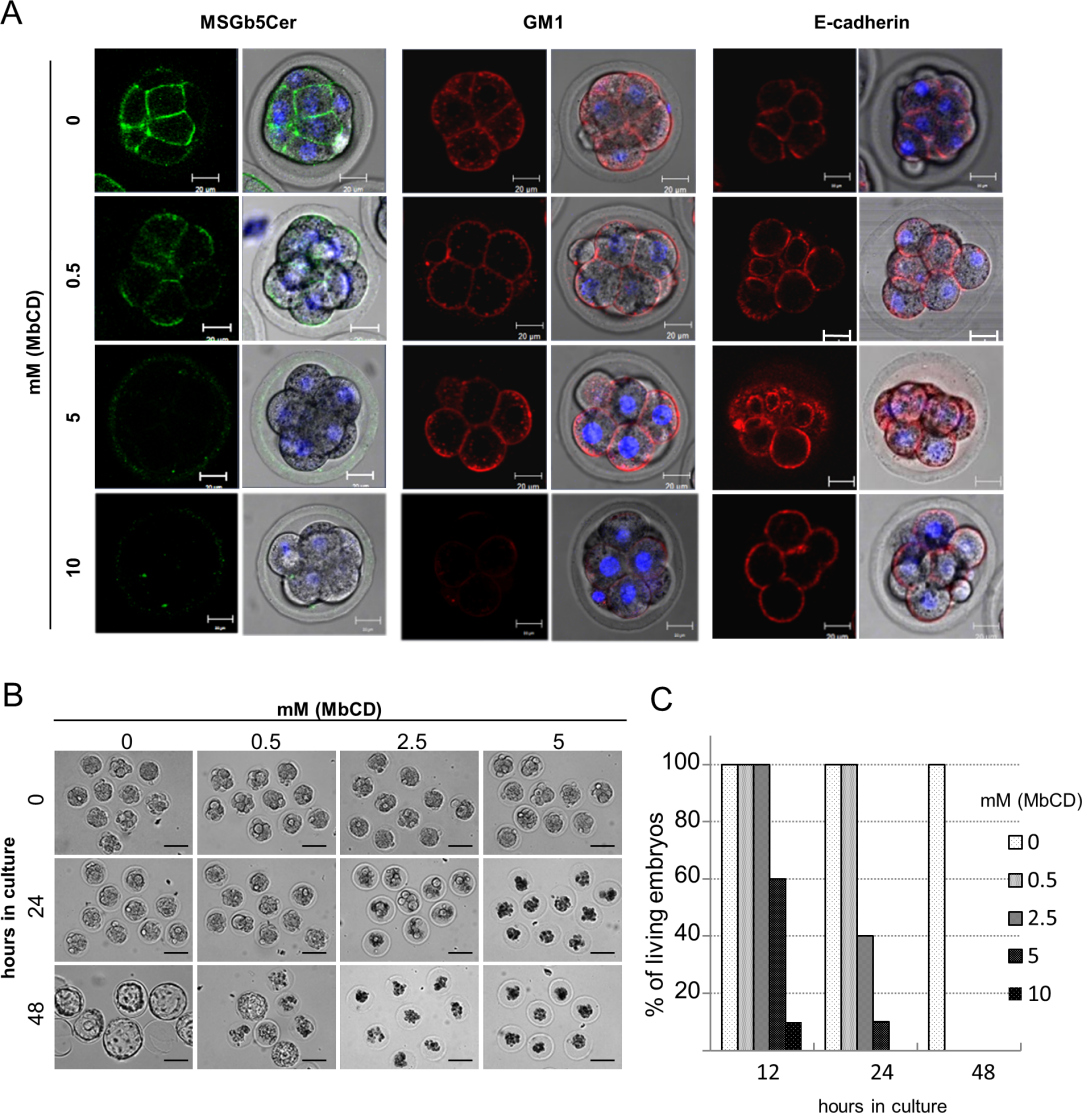
**Pretreatment with MbCD inhibits cytokinesis and development of mouse preimplantation embryos**. (A) Effect of MbCD on the expression of MSGb5Cer (green), GM1 (red) and E-cadherin (red) on 8-cell embryos. The left column and the right column of each panel represent a 2D image and the overlay of a 2D image and a differential interference contrast (DIC) micrograph, respectively. Thirty 8-cell embryos were used for the experiment represented in the images. (B) Representative DIC images of embryos cultured for 0, 24 and 48 hours in M16 medium after preincubation with 0-5 mM MbCD. Scale bars: 100 μm. Twenty 8-cell embryos were used for the experiment represented each images. (C) The percentages of living embryos cultured for 12, 24 and 48 hours after preincubation with 0-5 mM MbCD. Twenty 8-cell embryos were cultured for each concentration of MbCD.

Since cholesterol has been postulated to be an absolute requirement for raft integrity, MbCD, which depletes cholesterol from the cell surface, has been used as a tool in lipid raft research [13-15]. To investigate the correlation between raft integrity and the distribution of MSGb5Cer, GM1, and E-cadherin in the embryos, compacted 8-cell embryos were pretreated with various concentrations of MbCD for 15 minutes and then examined. As shown in Figure 4A, pretreatment of the embryos with 0.5 mM MbCD induced decompaction and decreased expression of MSGb5Cer at the interface between blastomeres. The expression on the outer membrane of blastomeres was not decreased and rather seemed to be increased. At a higher concentration (5 mM), expression of MSGb5Cer on the cell surface was further reduced, whereas expression of GM1 and E-cadherin was unaffected. The green fluorescence signals detected in the zona pellucida of 5 mM MbCD-treated embryos are thought to be derived from MSGb5Cer released from the embryo. Treatment with 10 mM MbCD caused complete loss of MSGb5Cer, and decreased expression of GM1, but there were no change in E-cadherin expression.

Next, we investigated the importance of raft integrity in viability of embryos. Pretreatment of 8-cell embryos with MbCD suppressed normal development in a time- and concentration-dependent manner (Figure 4B, C). All embryos in the control culture survived and developed normally into blastocysts by 48 hours of culture, whereas none of embryos pretreated with 0.5 mM MbCD had survived by 48 hours of culture. These results suggest that MSGb5Cer is more sensitive than GM1 to raft integrity, and that lipid raft is prerequisite to cell adhesion and normal development of preimplantation embryos.

### Extensive accumulation of MSGb5Cer to interfaces is related to delay and suppression of normal development

**Figure 5:**
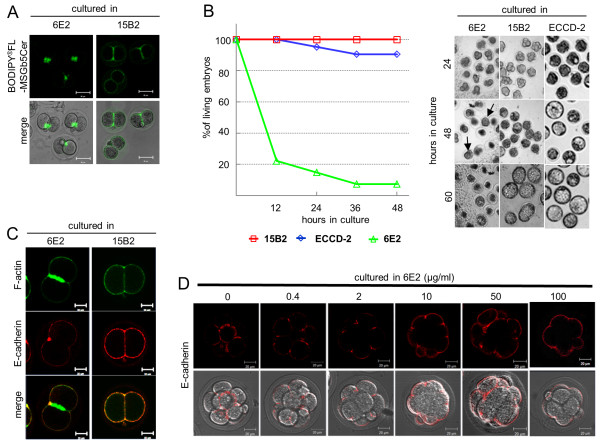
**Extensive cross-linking of MSGb5Cer with 6E2 inhibits cytokinesis and development of mouse preimplantation embryos**. (A) Deposition of BODIPY^®^FL-MSGb5Cer at the interfaces of 2-cell stage embryos cultured in the presence of 6E2. Embryos labeled with BODIPY^®^FL-MSGb5Cer (green) were cultured for 1 hour in M16 medium containing 100 μg/ml of 6E2 or 15B2 antibodies. A fluorescence 2D image of BODIPY^®^FL-MSGb5Cer and the overlay of a 2D image and DIC micrograph of cultured embryos are shown. These images shown are representative of twelve 2-cell embryos. (B) Effect of cross-linking of MSGb5Cer with 6E2 on cytokinesis and the development of mouse preimplantation embryos. Two-cell embryos were cultured for 60 hours in M16 medium containing 100 μg/ml of 15B2 (red squares), ECCD-2 (blue diamonds), and 6E2 (green triangles), and the living embryos were counted and their DIC images were taken at the times indicated. Arrows indicate compacted embryos. Twenty of 2-cell stage embryos were cultured for each experiment groups. (C) Localization of F-actin and E-cadherin in MSGb5Cer-cross-linked 2-cell embryos. Two-cell embryos treated for 2 hours with 6E2 (left column) or 15B2 (right column) were fixed and stained with Alexa Fluor^® ^633 phalloidin and anti E-cadherin/Alexa Fluor^® ^546-conjugated rabbit anti-rat IgG. These images shown are representative of twelve 2-cell embryos. (D) Inhibitory effect of cross-linking of MSGb5Cer with 6E2 on the localization of E-cadherin at interfaces. Embryos were immunostained with ECCD-2/Alexa Fluor^® ^546-conjugated Rabbit Anti-rat IgG (red) in the presence of various concentrations of 6E2. The upper row and the bottom row of each panel represent a 2D image and the overlay of a 2D image and DIC micrograph, respectively. These images shown are representative of thirty morula embryos.

As shown in Figure 5A, incubation for 1 hour in the presence of 100 μg/ml of anti- MSGb5Cer Mab 6E2, but not of the isotype-matched control Mab 15B2, led to extensive accumulation of MSGb5Cer at the interfaces in the embryos, and a large aggregate of BODIPY^®^FL-MSGb5Cer was found.

Prolonged culture of 2-cell embryos in the presence of 6E2 caused a decrease in survival rate, and more than 90% of the embryos died before developing into blastocysts (Figure 5B). 80% of embryos were died after 12 hour culture in 6E2, whereas all of embryos cultured in control Mabs were alive. After 24 hour-culture, embryos cultured in 6E2 cleaved abnormally, and their cleavage rate was delayed as compared with those cultured in control Mabs. The morula embryo, which was able to avoid being injured, seemed to be compacted (arrow in 48 hour culture in 6E2). The anti-MSGb5Cer Mab 6E2 we used in this study is not toxic and do not have non-specific effects on preimplantation embryos, because 100 μg/ml of 6E2 antibody does not affect viability of blastocyst stage embryos that no longer express MSGb5Cer (see Figure S1 in additional file 2). In addition, when either the isotype-matched control Mab 15B2 or anti-E-cadherin Mab ECCD-2 was similarly tested, they did not affect viability of embryos (Figure 5).

In the 2-cell embryos cultured for 2 hours with 6E2, a large amount of F-actin accumulated at the interfaces, whereas no E-cadherin was detected at the interfaces, and it almost localized on the outer surface of blastomeres (Figure 5C). The entry of E-cadherin into the interfaces in living embryos was inhibited after treatment with Mab 6E2 in a dose-dependent manner (Figure 5D).

### MSGb5Cer did not accumulate at the interfaces in actin-depolymerized 2-cell embryos

**Figure 6:**
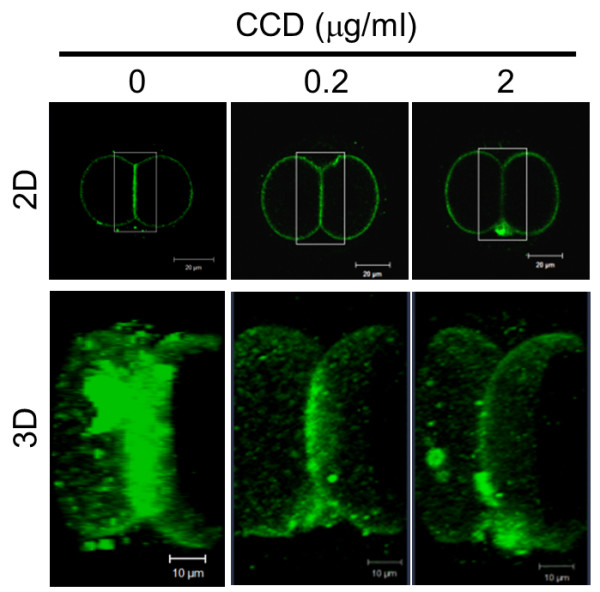
**Disruption of actin filaments suppresses localization of MSGb5Cer at the interface**. Two-cell embryos pretreated with 0, 0.2, and 2 μg/ml of CCD were immunostained with Alexa Fluor^® ^488-conjugated 6E2 (green). The 3D images were constructed by stacking optical slice images of the area enclosed by the square. Scale bar: 20 μm (2D), 10 μm (3D). These images shown are representative of thirty 2-cell stage embryos.

Actin filaments are thought to generate a mechanical force that drives membrane molecules or domains during cytokinesis. We investigated the effect of disruption of actin filaments with CCD on the localization of MSGb5Cer. As shown in Figure 6, when 2-cell stage embryos preincubated with 0, 0.2, and 2 μg/ml of CCD were immunostained with anti-MSGb5Cer Mab 6E2, accumulation of MSGb5Cer at the interface was suppressed in a dose-dependent manner. These results suggest that inhibition of F-actin polymerization by CCD prevents the localization of MSGb5Cer at the interface.

### Absence of E-cadherin causes heterotopic localization of MSGb5Cer on the blastomere surface

**Figure 7:**
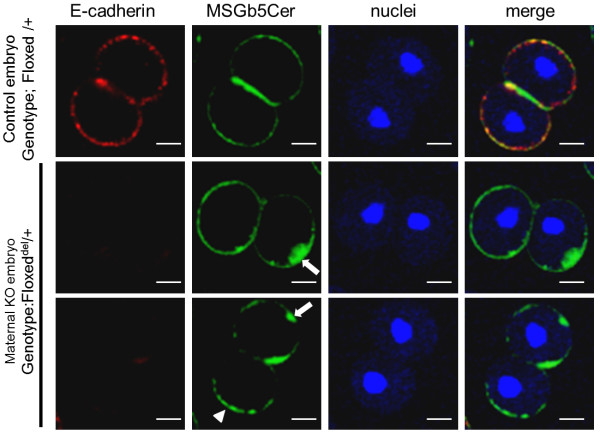
**Absence of E-cadherin causes heterotopic localization of MSGb5Cer on the blastomere surface**. Embryos expressing E-cadherin (Genotype; Floxed/+) and lacking E-cadherin (Genotype; Floxed^del^/+) were dually immunostained with ECCD-2/Alexa Fluor^® ^546-conjugated Rabbit Anti-rat IgG and Alexa Fluor^® ^488-conjugated 6E2. 2D images of E-cadherin (red), MSGb5Cer (green) and nuclei (blue) are merged in the far right column. We used thirty maternal knockout embryos and seventeen control embryos in this experiment. MsGb5Cer represents heterotopic localization in all maternal knockout embryos.

To investigate the involvement of E-cadherin in the localization of MSGb5Cer at the interface, we generated embryos lacking maternal E-cadherin, and examined them. In control 2-cell embryos (Genotype; Floxed/+), MSGb5Cer and E-cadherin exhibited a similar distribution pattern and accumulated at the interface (Figure 7 upper row). In E-cadherin null mutant 2-cell embryos (Genotype; Floxed del/+), adhesion between the blastomeres was weaker and the area of the interface plane was greatly reduced (Figure 7 middle and bottom row). In these embryos, MSGb5Cer formed heterotopic aggregates (indicated by the arrow) or assembled at the outer surface membranes of the blastomeres (indicated by the arrowhead). These results suggest that E-cadherin is required not for assembly, but for localization of MSGb5Cer at the interface of blastomeres.

## Discussion and Conclusions

In this study, we investigated the involvement of MSGb5Cer in the development of mouse preimplantation embryos. As presented in Figure 2, MSGb5Cer moved to the future site of the cleavage furrow and accumulated at the interfaces between blastomeres as cytokinesis proceeds during embryonic development of mouse preimplantation embryos. Once, however, cytokinesis is complete the MSGb5Cer have accumulated at the interfaces evenly redistribute themselves over the cell surface (Figure 2).

MSGb5Cer is thought to be involved in the formation of raft membrane domains. Indeed, we observed overlapped localization of MSGb5Cer and cholesterol, an important constituent of lipid rafts (Figure 3). The finding that disruption of the integrity of lipid rafts by removal of cholesterol from the cell membrane with MbCD caused the release of MSGb5Cer into the perivitelline space (Figure 4) should further support above notion. Interestingly, as we presented in this study (Figure 1), GM1 and MSGb5Cer localize differently, suggesting that they may belong to different lipid rafts that possess distinct functions as described in several studies [16-18].

Since GM1 is easily detected with CTX-B, it is considered one of the most important marker GSLs for lipid rafts. Comiskey and Warner reported observation that GM1 visualized with biotinylated CTX-B were enriched at the cleavage furrow in mouse 2-cell and 4-cell embryos which actively undergo cytokinesis [10]. Burgess DR and his co-workers also reported that plasma membrane domains enriched in GM1 contain signalling machinery that contributes to cytokinesis and accumulate in the equatorial plasma membrane at mid-anaphase in sea urchin eggs [9], and further revealed that cells are polarized upon insertion of distinct basolateral membrane at the first division using the apical marker GM1 and the aPKC-PAR6 complex [19]. Although GM1 is constantly expressed throughout the cleavage stage in preimplantation embryos, MSGb5Cer is expressed only in the early cleavage stage from the unfertilized egg stage to morula stage embryos and more abundantly enriched at the cleavage furrow than GM1 during the course of cytokinesis (Figure 1). MSGb5Cer should therefore play a more specific role as a constituent of lipid rafts at this stage.

As described above, MbCD-mediated disruption of the integrity of lipid rafts caused the release of MSGb5Cer into the perivitelline space and decompaction of compacted 8-cell embryos and suppressed cell division. Comiskey and Warner also showed that cholesterol depletion by treatment of zygotes with MbCD inhibits preimplantation development from zygotes to blastocysts stage in culture in a dose-dependent manner [10]. On the other hand, heavy cross-linking of MSGb5Cer with MSGb5Cer-specific Mab induces extensive aggregation of MSGb5Cer (Figure 5) and suppressed cytokinesis. As a consequence, normal embryonic development would be terminated in both cases. Therefore, lipid rafts enriched in MSGb5Cer should play an important role in cytokinesis as well as in embryogenesis.

During cytokinesis, the cortical actin network form a scaffold for membrane proteins and thereby transfer them toward the cleavage furrow [3]. E-cadherin knockout mice display embryonic lethality and embryos are unable to form adhesion complexes [20,21]. In this study, we also presented close correlations between lipid rafts enriched in MSGb5Cer and E-cadherin as well as the cortical actin network. In preimplantation embryos, MSGb5Cer and E-cadherin are similarly distributed at the interfaces between blastomeres in 8-cell embryos, while MbCD-treatment caused the release of MSGb5Cer into the perivitelline space, decompaction of compacted embryos and even distribution of E-cadherin on the cell surface. In 2-cell embryos lacking E-cadherin, however, the MSGb5Cer-enriched lipid rafts accumulated heterotopically at sites other than the interface, indicating a close association with E-cadherin in maintaining the integrity of MSGb5Cer movement. On the other hand, disruption of polymerization of F-actin with CCD dose-dependently inhibited accumulation of MSGb5Cer at the interfaces, suggesting that the movement of MSGb5Cer-enriched lipid rafts into interfaces is driven by F-actin. During extensive aggregation of MSGb5Cer induced by cross-linking with specific antibody, all of the F-actin accumulated at the interfaces.

Although the details are still remaining to be clarified and further experiments are clearly needed, these results suggest that MSGb5Cer-enriched lipid rafts are driven into the furrow by the cortical actin network in close association with E-cadherin and play a role in cytokinesis in the early cleavage stage of mouse preimplantation embryos and our findings in this study should provide clues to the functional role of lipid rafts in early embryogenesis.

## Methods

### Embryo collection and culture

In all experiments, 6-8 week old female BDF1 mice purchased from CLEA Japan. Inc. (Tokyo, Japan) were induced to superovulated by intraperitoneal injections of pregnant mares' serum (ASKA Pharmaceutical Co., Ltd., Japan), and of human chorionic gonadotropin (hCG; ASKA Pharmaceutical Co., Ltd.) 48 hours later. Immediately after the hCG injection, each female mouse was mated with a male of the same strains. Zygotes and 2-cell and 8-cell embryos were collected by flushing out of the oviducts into M16 medium at 24 hours, 36 hours, and 60 hours, respectively, after the hCG injection and then cultured at 37°C in a conventional incubator. Animals were treated according to the institutional animal care and use guidelines of the National Research Institute for Child Health and Development. To obtain embryos lacking maternal E-cadherin, C57BL/6 mice were crossed as shown in Figure S2 in additional file 3 according to the procedure described by De Vries et al. [21]. The embryos did not express E-cadherin translated from paternal transcripts, at least until the 2-cell stage. Genotyping of all mice was performed by PCR using DNA extracted from ear snips of 28-day-old mice with an Automatic DNA Extraction System (NA-3000, KURABO, Tokyo, Japan). The following primer pair specific for cre recombinase was used to determine whether the Zp3-cre transgene was present: Cre1 (5'-ATG CCC AAG AAG AAG AGG AAG GT-3'), Cre2 (5'-GAA ATC AGT GCG TTC GAA CGC TAG A-3'). The primer pairs used to detect the different alleles of E-cadherin were as described previously by Boussadia et al. [22].

### Antibodies and chemicals

Alexa Fluor^® ^488-conjugated 6E2, used for staining MSGb5Cer, was prepared as previously described [11]. Anti-mouse E-cadherin Mab ECCD-2 was purchased from TAKARA Bio. Co. (Tokyo Japan). Mab 15B2 was a generous gift of Dr. Taketo Yamada of Keio University School of Medicine [23]. Alexa Fluor^® ^633 phalloidin and Alexa Fluor^® ^546 CTX-B, used for staining actin and GM1, respectively, were purchased from Invitrogen. Alexa Fluor^® ^546 Rabbit Anti-rat IgG and Alexa Fluor^® ^546 streptavidin were also purchased from Invitrogen. Biotinylated-CTX-B of Sigma-Aldrich Inc. (St. Louis. MO) was also used. Filipin III, used for staining cholesterol, was purchased from Cayman Chem. Co. (Ann Arbor, MI). MbCD and CCD were obtained from Sigma and Calbiochem, respectively. GM1 and a monosialylganglioside mixture were purchased from Matreya Inc. (Pleasant Gap, PA).

### Immunostaining of mouse preimplantation embryos

Immunostaining of "living" mouse preimplantation embryos was performed as previously described [11]. Briefly, cells were incubated in 30 μl drops of M16 medium containing 0.45 μg of Alexa Fluor^® ^488 or 546 -conjugated 6E2 (final concentration 15 μg/ml), and then they were washed three times in 30 μl drops of M16 medium. All staining steps were carried out at 37°C in a CO_2 _incubator for fresh embryos. Cell nuclei were stained with DAPI (Invitrogen), which slowly permeates the living cell membrane and slowly leaks out after washing [24]. For actin and cholesterol staining, embryos were prefixed for 10 minutes at room temperature with 2% paraformaldehyde containing 0.1% glutaraldehyde in 4-(2-hydroxyethyl)-1-piperazineethanesulfonic acid (HEPES) buffered saline (HBS), then permeabilized with 0.01% Triton X-100 in HBS for 10 minutes at room temperature, and blocked with 3% bovine serum albumin. Staining of living embryos and fixed embryos was performed at 37°C and 4°C, respectively. The stained embryos were placed in a microdrop of M16 medium on a glass-bottom dish (IWAKI Glass, Tokyo, Japan), covered with liquid paraffin (NAKALAI TESQUE, Kyoto, Japan), and examined with a LSM510 Zeiss Confocal laser-scanning microscope (Carl Zeiss, Thornwood, NY) and a 40× objective lens so that only the embryo was included in the field of view. For three dimensional (3D) construction, two-dimensional (2D) images were captured as vertical sections (at approximately 2-μm intervals) by using a Z-axis motor, then processed with Zeiss Zen 2009 software (Carl Zeiss), and finally stacked into one picture.

### Preparation of BODIPY^®^FL-MSGb5Cer

MSGb5Cer was purified from human renal cancer cell line ACHN cells by ion-exchange chromatography on DEAE-Sephadex A25 and preparative TLC/TLC blotting according to Taki et al. MSGb5Cer was conjugated to fluorescence reagents with Sphingolipid Ceramide N-deacylase (SCDase) according to the procedure described in the previous report [25]. To prepare lyso-MSGb5Cer, MSGb5Cer was incubated at 37°C for 16 hours with 8 μU of SCDase in 20 μl of 25 mM sodium acetate buffer, pH5.5, containing 0.2% Triton X-100 and 5 mM CaCl_2_, and the reaction mixture was adsorbed onto an Oasis^® ^MCX cartridge (Waters). The lyso-MSGb5Cer eluted from the cartridge with 5% ammonium hydroxide in methanol was subsequently incubated with BODIPY^®^FL-C12 (Invitrogen) in 20 μl of the condensation reaction mixture (8 mU SCDase, 25 mM Tris-HCl buffer, pH7.5, 5 mM MgCl_2_, 0.1% Triton X-100) at 37°C for 16 hours. The reaction mixture was adsorbed to an Oasis MCX cartridge. The methanol eluate was dried, desalted with a DISCOVERY DC-18 cartridge (SUPELCO), and adsorbed to a DISCOVERY DSC-Si cartridge (SUPELCO). The condensed BODIPY^®^FL-MSGb5Cer product eluted from the cartridge with methanol was analyzed by high performance thin layer chromatography (HPTLC) by using chloroform/methanol/0.02% CaCl_2 _(5:4:1, v/v) as the developing solvent, and it was viewed by using FLA-7000 (Fuji Film, Tokyo, Japan) (see Figure S3 in additional file 4). BODIPY^®^FL-GM1 was also prepared in the same manner as described above. MSGb5Cer and GM1 enzymatically conjugated with BODIPY^®^FL-C12 -fatty acid in ethanol were dissolved in 3.4% non-fatted BSA in Hank's balanced solution and diluted in M16 medium. Two-cell stage embryos were incubated in a drop of the medium for 20 minutes and examined.

### Time-lapse imaging

MSGb5Cer was stained by incubating the zygote at 22-26 hours after the hCG injection for 20 minutes in a conventional incubator with Alexa Fluor^® ^488-conjugated 6E2 Mab and DAPI dissolved in M16 medium. Stained embryos were transferred to a microdrop of M16 medium on a glass-bottom dish covered with liquid paraffin. During time-laps imaging, samples were placed in a 37°C stage-top incubator (INUBG2-PPZI, Tokai Hit, Japan) with a slow flow of air with 5% CO2 gas and high humidity. An objective heater (INUBG2-PPZI, Tokai Hit, Japan) was also used to maintain the objective temperature at 37°C to reduce temperature gradients within the sample. Time-lapse fluorescence images were taken at 15-minute intervals with a spinning disk confocal scanhead (Yokogawa) attached to an inverted fluorescence microscope (OLYMPUS). Images were captured as vertical sections (approximately 2-μm intervals) by using a Z-axis motor and processed by a deconvolution program using iQ software (Andor). Fluorescence intensity was measured with ImageJ software http://rsb.info.nih.gov/ij/.

### Pretreatment of embryos with MbCD

A stock solution of MbCD was prepared in sterile water at 100 mM and diluted with M16 medium. Compacted embryos were preincubated with 0, 0.5, 2.5, 5, and 10 mM MbCD for 15 minutes, and then cultured for 48 hours. The embryos after preincubation with MbCD were immunostained with Alexa Fluor^® ^488-conjugated 6E2, Alexa Fluor^® ^546 CTX-B, and ECCD-2/Alexa Fluor^® ^546 conjugated-rabbit anti-rat IgG to examine the distribution of MSGb5Cer, GM1, and E-cadherin, respectively.

### Embryo culture in the presence of anti-MSGb5Cer Mab

Two-cell stage embryos were cultured for 48 hours in M16 medium containing 100 μg/ml of anti-MSGb5Cer Mab 6E2, ECCD-2, or 15B2. ECCD-2 was used as a control Mab that binds to mouse embryos. 15B2 was used as a control Mab subclass-matched to 6E2 that do not binds to mouse embryos. The aliquots of embryos after culture for 2 hours in the presence of 6E2 or 15B2 were fixed and immunostained with Alexa Fluor^® ^633 phalloidin and ECCD-2/Alexa Fluor^® ^546 Rabbit Anti-rat IgG to examine the distribution of F-actin and E-cadherin, respectively.

### Pretreatment of embryos with CCD

A stock solution of CCD was prepared in DMSO at 2 mg/ml and diluted with M16 medium. Two-cell stage embryos were preincubated for 30 minutes at 37°C with 0, 0.2, and 2 μg/ml of CCD in a CO_2 _incubator. After washing with M16 medium, the embryos were immunostained for 1 hour with Alexa Fluor^® ^488-conjugated 6E2.

## List of abbreviations

GSL: Glycosphingolipid; MSGb5Cer: MonosialylGb5ceramide; MbCD: Methyl-beta-cyclodextrin; CCD: Cytochalasin D; Mab: Monoclonal antibody; SSEA-4: Stage-specific embryonic antigen-4; CTX-B: Cholera toxin B subunit; hCG: human chorionic gonadotropin; HEPES: 4-(2-hydroxyethyl)-1-piperazineethanesulfonic acid; HBS: HEPES buffered saline; HPTLC: high performance thin layer chromatography; SCDase: Sphingolipid Ceramide N-deacylase; DIC: differential interference contrast.

## Authors' contributions

BS designed and carried out most of experiments and drafted the manuscript. YUK, KT and NK designed the experiments and edited the manuscript. KM, HA, JF and AU helped conceive the experiments and commented on the manuscripts. NO and MI helped in the preparation of fluorescence labeled-glycosphingolipid. JF and NK obtained funding. All authors read and approved the final manuscripts.

## Supplementary Material

Additional file 1 **MSGb5Cer accumulates at the interface during cytokinesis**. 6E2 stained cells were imaged by confocal microscopy during mitosis. Frames were taken every 15 minutes and are displayed at 2 frames per second. Click here for file

Additional file 2 **The data of blastocysts culture in the presence of 100 μg/ml of 6E2**. Click here for file

Additional file 3 **Mating scheme used to generate embryos lacking E-cadherin**. Click here for file

Additional file 4 **HPTLC showing the preparation of BODIPY^®^FL-GM1 and -MSGb5Cer**. Click here for file
