# Supplementary figures and images for: Lipid rafts enriched in monosialylGb5Cer carrying the stage-specific embryonic antigen-4 epitope are involved in development of mouse preimplantation embryos at cleavage stage
Source: BMC Dev Biol. 2011 Apr 14;11:22. doi: 10.1186/1471-213X-11-22 (PMC3089780; doi:10.1186/1471-213X-11-22)

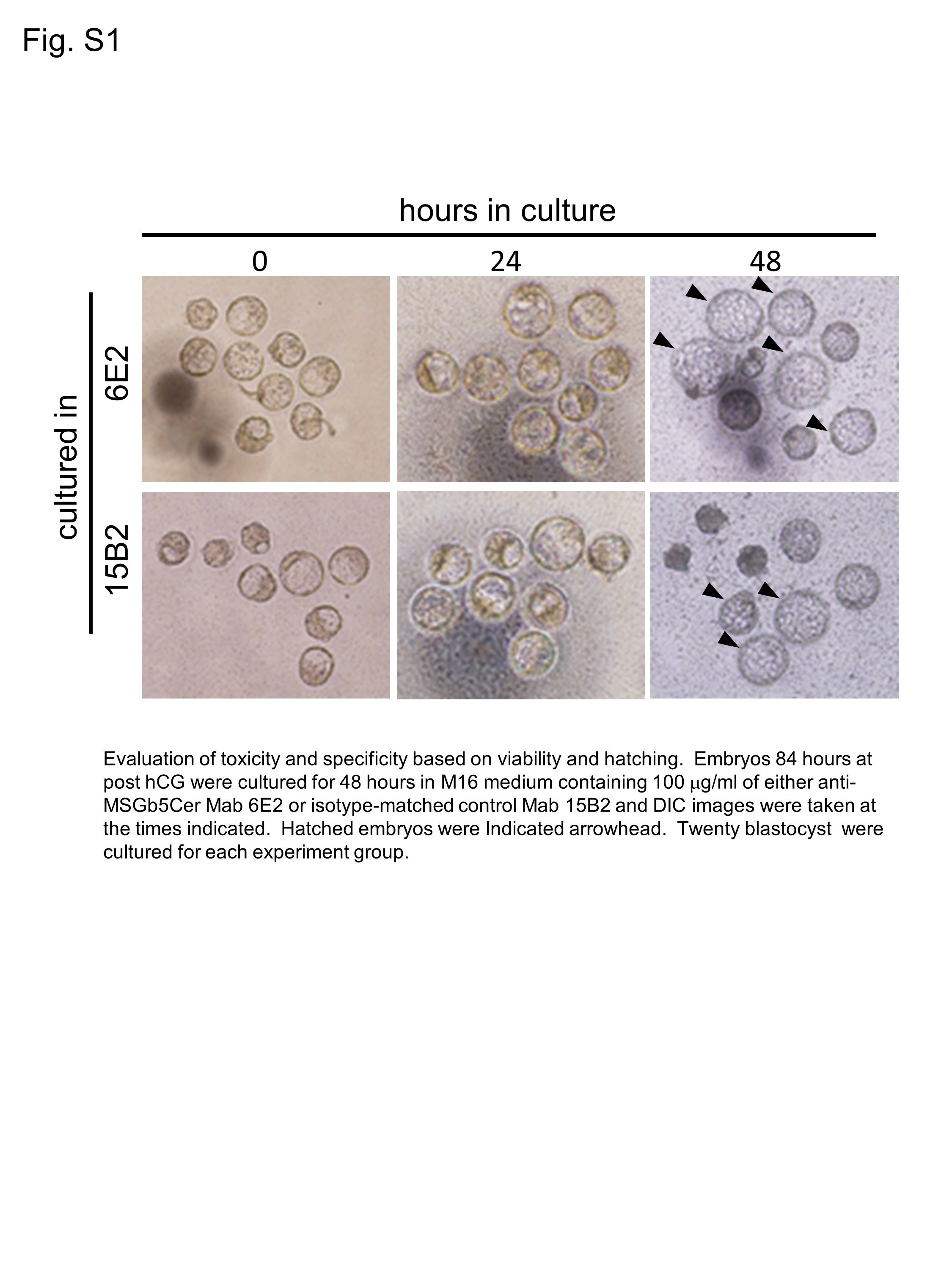

Supplement: Additional file 2 — The data of blastocysts culture in the presence of 100 μg/ml of 6E2. [file 1471-213X-11-22-S2.TIFF]

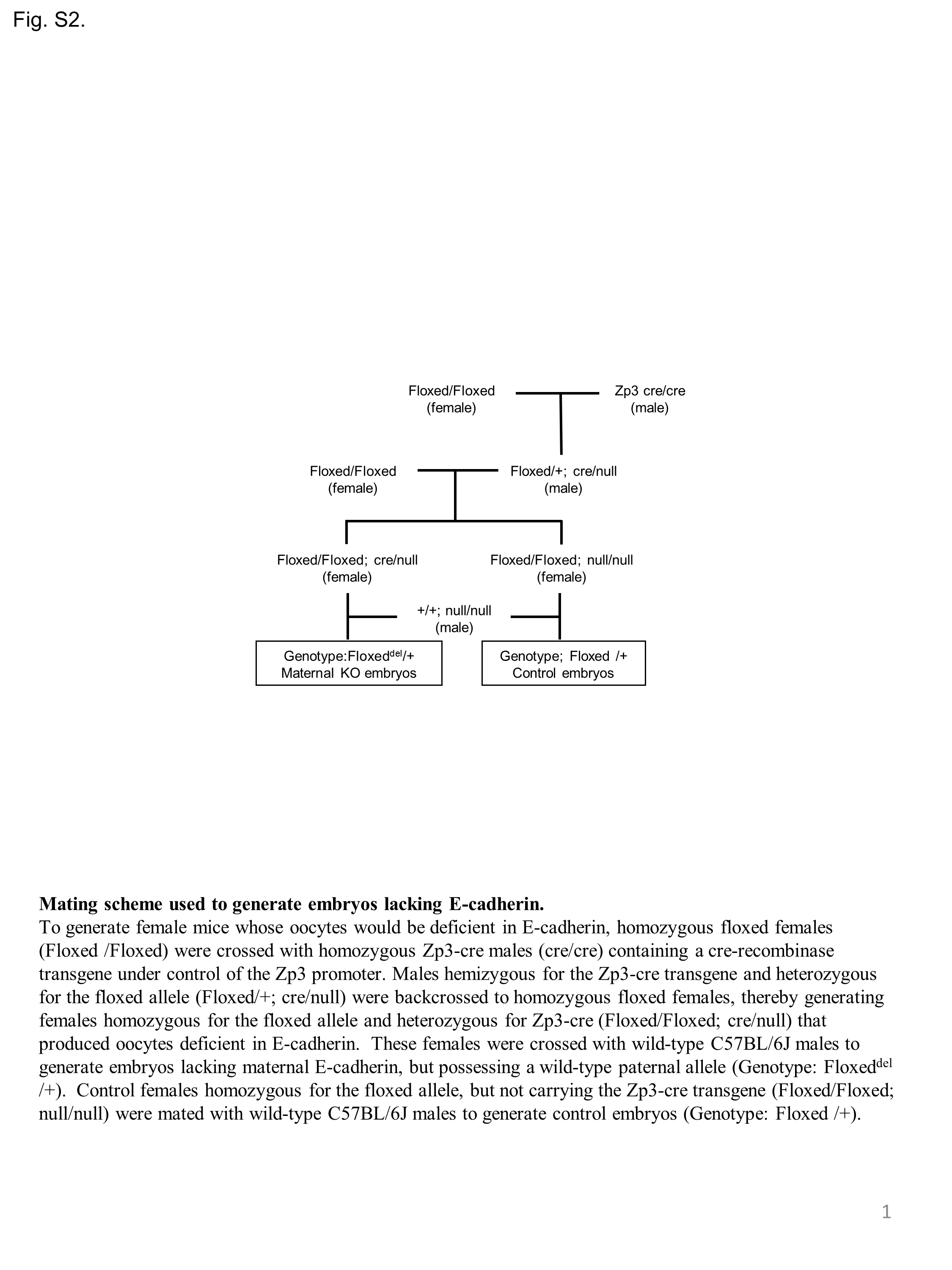

Supplement: Additional file 3 — Mating scheme used to generate embryos lacking E-cadherin. [file 1471-213X-11-22-S3.TIFF]

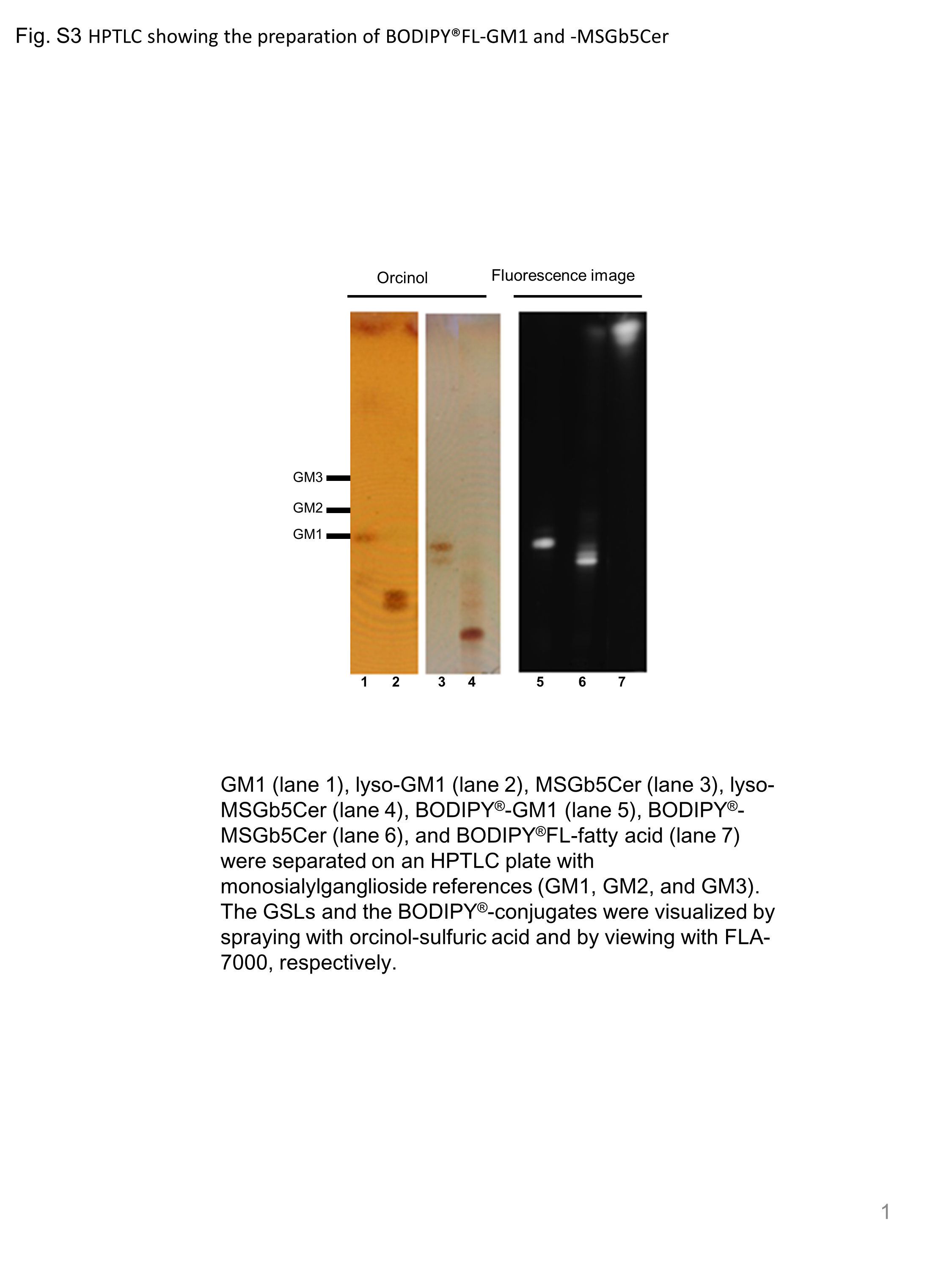

Supplement: Additional file 4 — HPTLC showing the preparation of BODIPY®FL-GM1 and -MSGb5Cer. [file 1471-213X-11-22-S4.TIFF]
